# Alveolar epithelial cells undergo epithelial-mesenchymal transition in acute interstitial pneumonia: a case report

**DOI:** 10.1186/1471-2466-14-67

**Published:** 2014-04-23

**Authors:** Hongbo Li, Jinjin Zhang, Xiaodong Song, Tao Wang, Zhi Li, Dong Hao, Xiaozhi Wang, Qingyin Zheng, Cuiping Mao, Pan Xu, Changjun Lv

**Affiliations:** 1 Department of Pulmonary medicine, Binzhou Medical University Hospital, Binzhou, P.R. China; 2 Medicine Research Center, Binzhou Medical University, Yantai, P.R. China; 3 Department of Otololaryngology, Case Western Reserve University, Cleveland, OH, USA

**Keywords:** Acute interstitial pneumonia, Epithelial-mesenchymal transition, Myofibroblast

## Abstract

**Background:**

Acute interstitial pneumonia is a rare interstitial lung disease that rapidly progresses to respiratory failure or death. Several studies showed that myofibroblast plays an important role in the evolution of diffuse alveolar damage, which is the typical feature of acute interstitial pneumonia. However, no evidence exists whether alveolar epithelial cells are an additional source of myofibroblasts via epithelial-mesenchymal transition in acute interstitial pneumonia.

**Case presentation:**

In this report, we present a case of acute interstitial pneumonia in a previously healthy 28-year-old non-smoking woman. Chest high-resolution computed tomography scan showed bilateral and diffusely ground-glass opacification. The biopsy was performed on the fifth day of her hospitalization, and results showed manifestation of acute exudative phase of diffuse alveolar damage characterized by hyaline membrane formation. On the basis of the preliminary diagnosis of acute interstitial pneumonia, high-dose glucocorticoid was used. However, this drug showed poor clinical response and could improve the patient’s symptoms only during the early phase. The patient eventually died of respiratory dysfunction. Histological findings in autopsy were consistent with the late form of acute interstitial pneumonia.

**Conclusions:**

The results in this study revealed that alveolar epithelial cells underwent epithelial-mesenchymal transition and may be an important origin of myofibroblasts in the progression of acute interstitial pneumonia. Conducting research on the transformation of alveolar epithelial cells into myofibroblasts in the lung tissue of patients with acute interstitial pneumonia may be beneficial for the treatment of this disease. However, to our knowledge, no research has been conducted on this topic.

## Background

Acute interstitial pneumonia (AIP), also known as Hamman-Rich syndrome, is a fulminating interstitial lung disease characterized by acute respiratory failure. The clinical features presented by majority of patients are described as a flulike prodrome including sore throat, headache, cough, dyspnea, and often fever with abrupt onset and short duration [1].

The histological hallmark of AIP was defined as diffuse alveolar damage (DAD), which is a nonspecific reaction in the lung to many injurious agents. The pathologic progress of DAD can be separated into three phases: acute exudative phase, which is characterized by interstitial edema, hyaline membrane, and acute interstitial inflammation accumulation [2]; proliferative phase, which is characterized by interstitial thickening and the appearance of granulation tissue in alveolar spaces [3]; and fibrotic phase, which is characterized by enlarged fibrotic septa and laminated intra-alveolar fibrosis [4].

The primary focus of therapy is supportive care. However, the use of glucocorticoids and immunosuppressive therapies is only effective in some cases. The case-fatality ratio remains high (>60 percent) despite intensive treatment and the majority of patients die within six months of presentation [5]. Thus, the pathologic process of the disease should be urgently explored, and a new therapeutic target should be identified.

Epithelial-mesenchymal transition (EMT), defined by the loss of epithelial characteristics and the acquisition of a mesenchymal phenotype, is essential for the progress of embryonic development [6]. Numerous studies revealed that the abnormal activation of EMT programs plays an important role in tissue fibrosis, cancer invasion, and metastasis [7-9]. However, the emergence and importance of EMT in lungs of patients with AIP remain unclear.

In this report, we present the case of a 28-year-old female diagnosed with AIP through histological and radiological lung examinations. Pathological and ultrastructural findings at open lung biopsy and autopsy showed that alveolar epithelial cells underwent EMT which may be beneficial for early intervention of AIP.

## Case presentation

**Figure 1:**
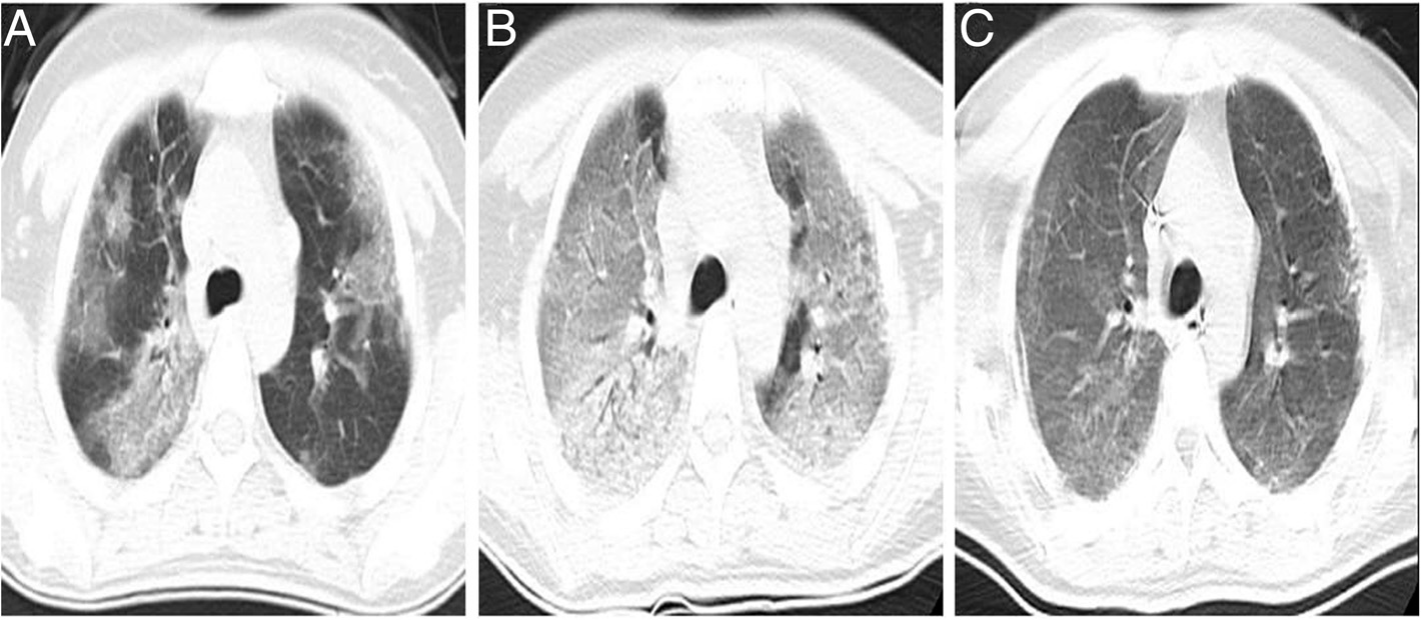
**Radiological findings of the patient. A**, Chest High-resolution computed tomography (HRCT) showed bilateral diffuse airspace opacification when the patient was hospitalized; **B**, Chest HRCT, taken on the fourth hospital day, demonstrated the worsening of diffused ground-glass opacification. **C**, the HRCT findings of the patient, who was treated with high dose of methylprednisolone for 5 d, showed diffuse ground-glass attenuation.

**Figure 2:**
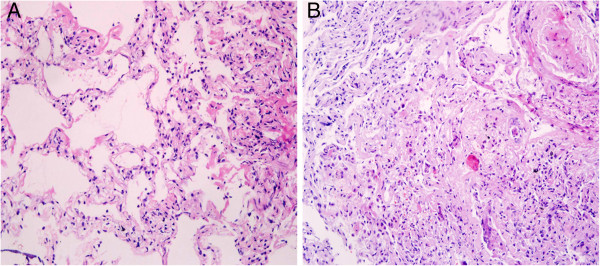
**Hematoxylin and eosin (H & E) stain. A**, Lung biopsy revealed edema, hyaline membranes, and acute interstitial inflammation representing the exudative phase of DAD. **B**, Lung autopsy showed interstitial and intra-alveolar fibroblast proliferation, which is the feature of the fibrotic phase.

**Figure 3:**
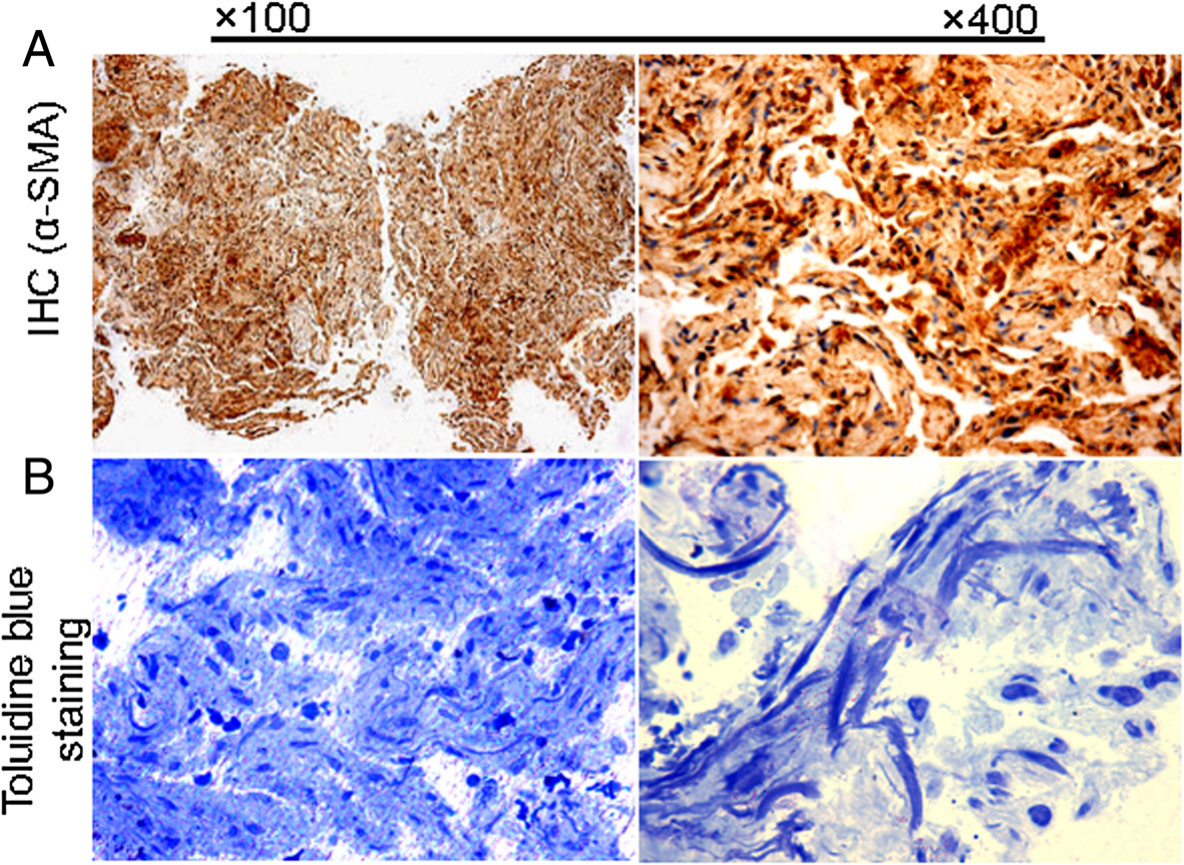
**Emergence of myofibroblasts in lung tissue. A**, Immunohistochemistry staining showed that an amount of α-SMA-positive cells existed in the lung section from autopsy (62 d). **B**, Toluidine blue staining demonstrated a significant amount of fibroblastic foci in the lung section from autopsy (62 d).

**Figure 4:**
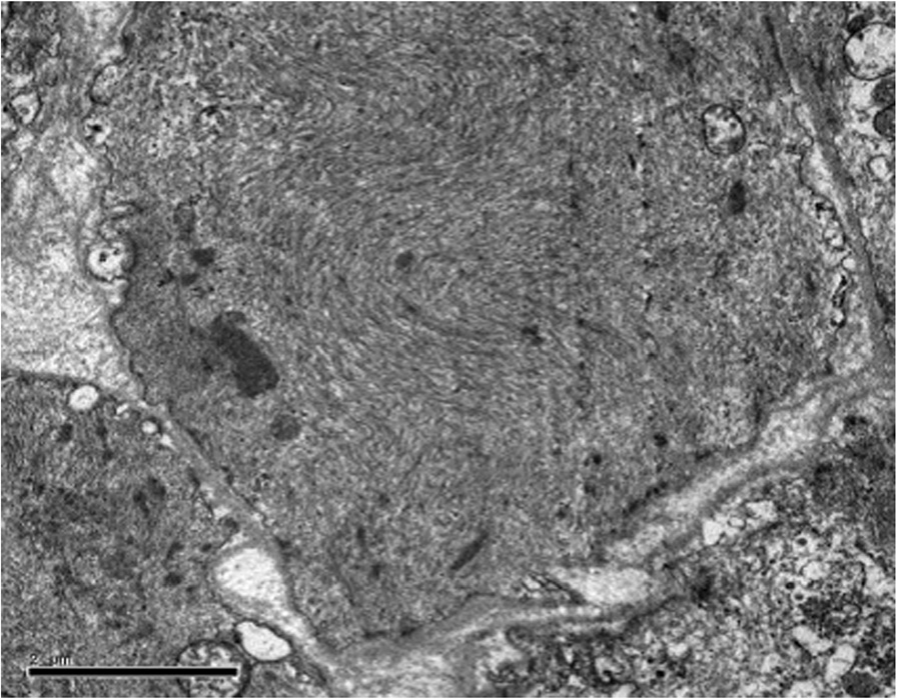
**Ultrastructural findings of the lung tissue from the patient.** Ultrastructural finding on autopsy (62 d) of the lung revealed bundles of actin microfilaments in the epithelial cells characterized partly by basement membrane.

**Figure 5:**
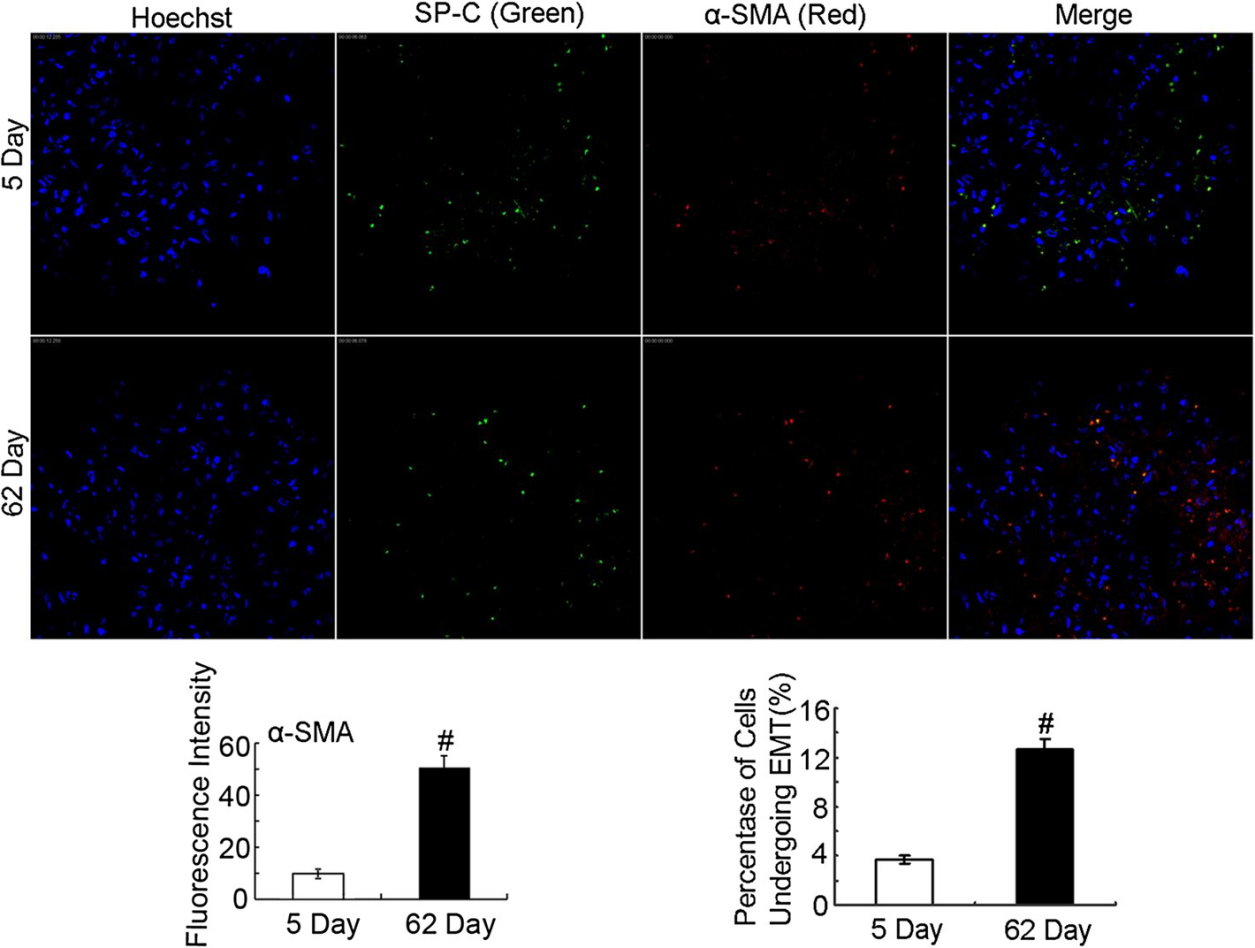
**Double positive cells for SPC and α**-**SMA existed in the human lung tissue**. The lung sections from biopsy (5 d) and autopsy (62 d) were stained with SPC (green) and α-SMA (red) antibodies followed by incubation with fluorescent dye-tagged secondary antibodies. Hoechst was used to stain the nuclei (blue). The mean fluorescence intensity of α-SMA in each whole image was automatically quantified by Image-Pro Plus software and expressed in fluorescence units (FU) and bar graph represents the average value from three independent experiments (left). Yellow fluorescence represents fluorescence coexpression indicating that epithelial cells underwent EMT. The α-SMA^+^SPC^+^ cells and SPC^+^ cells were counted respectively in each whole image and bar graph represents the average value (α-SMA^+^SPC^+^ cells/ SPC^+^ cells) from three independent experiments (right). P-values were calculated using two-tailed Student’s t-tests. ^#^, P < 0.05.

**Figure 6:**
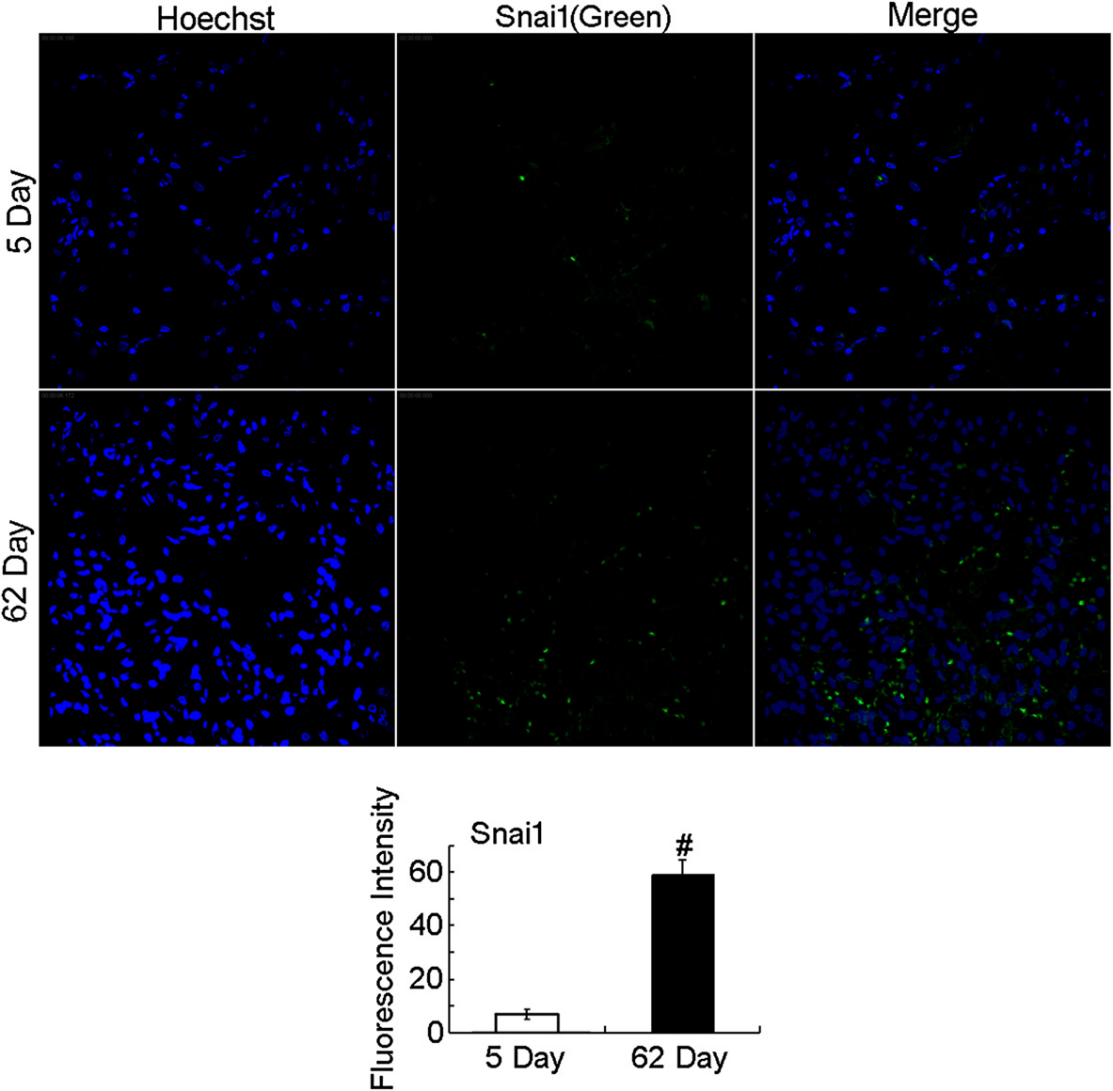
**Detection of Snai1 expression in human lung tissue.** The EMT transcription factor was detected by confocal immunofluorescence microscopy. The mean fluorescence intensity of Snai1 in each whole image was automatically quantified by Image-Pro Plus software and expressed in fluorescence units (FU). Bar graph represents the average value from three independent experiments. Increased expression of Snai1 (green) was observed in the lung sections from biopsy (5 d) and autopsy (62 d). P-values were calculated using two-tailed Student’s t-tests. ^#^, P < 0.01.

A previously healthy 28-year-old non-smoking woman was admitted to the hospital because of cough, mild dyspnoea, and fever of 38°C. The blood pressure, heart rate, and respiration rate of the patient were 103.0/63.0 mmHg, 100 beats/min, and 25 beats/min, respectively. During physical examination, she presented tachycardia, cyanosis, and diffusely reduced breath sounds but no vesicular murmur, crackles, or wheezing. Blood gas analysis revealed the following findings: pH 7.47; pCO_2_, 40 mmHg; pO_2_, 53 mmHg; HCO_3_^-^, 29.1 mmol/L; and Lac, 0.6 mmol/L. These findings indicate hypoxemia. High-resolution computed tomography (HRCT) of the chest revealed bilateral diffuse airspace opacification (Figure 1A). Levofloxacin was administered intravenously for 4 d. Her condition deteriorated with acute onset of dyspnoea and rapidly progressive respiratory failure, and the patient required intubation and mechanical ventilation. Blood gas analysis indicated hypoxemia; pH 7.19; pO_2_, 32 mmHg; and pCO_2_, 35 mmHg. HRCT revealed the deterioration of diffuse ground-glass opacification (Figure 1B). Fiberoptic bronchoscopy was performed on the same day after the patient was transferred to an intensive care unit. The bronchial tubes were normal with little sputum. Microbiologic investigations were negative. Transbronchial lung biopsies were performed on the same lobe (left upper lobe) on the fifth day of hospitalization. The first lung specimen exhibited edema, hyaline membrane formation, and acute interstitial inflammation, which all suggest an exudative phase of AIP (Figure 2A). High doses of intravenous methylprednisolone (500 mg for 3 d and 160 mg for 2 d) were administered based on presumptive diagnosis of interstitial lung disease. After 5 d, HRCT revealed diffuse ground-glass attenuation (Figure 1C). However, the patient still had acute hypoxic respiratory failure and could not be extubated after the procedure. Despite initiating intravenous with normal dose of methylprednisolone (80 mg per day) and high concentrations of oxygen, the patient remained intubated for eight weeks. Chest X-ray analysis indicated that her lung deteriorated did not improve even when high-dose glucocorticoid was re-administered. Her oxygen saturation was only 30% when the fraction of inspired oxygen was 100%. The patient eventually expired after 62 d of hospitalization. The patient underwent autopsy, and the slides showed diffusely thickened alveolar septal interstitium by hyaline membrane remnants, pulmonary interstitial and alveolar edema, type II pneumocyte hyperplasia (Figure 2B), and significant amount of myofibroblasts (Figure 3). These results indicate organized DAD. Transmission electron microscopy revealed that the epithelial cells, which are characterized by their basement membrane and specialized cell-cell junction structures, contained bundles of filament in the cytoplasm (Figure 4). The presence of filaments in these cells suggested the occurrence of EMT. We then determined the changes in EMT-related proteins, such as surfactant associated protein (SPC), α-smooth muscle actin (α-SMA), and Snail. (Santa Cruz Biotechnology Inc. Dallas, Texas, USA). The results of the double immunostaining of the lungs showed mesenchymal specific protein α-SMA in the alveolar epithelial cells (SPC-positive cells). The double positive cells were evidently increased in the lung tissue from the 62 d autopsy compared with that from the 5 d biopsy (Figure 5). The zinc finger transcription factor Snail was evidently expressed in the lung tissue, especially in the lung tissue from the 62 d autopsy (Figure 6).

## Conclusions

AIP is one of the six subtypes of major idiopathic interstitial pneumonia according to the American Thoracic Society/European Respiratory Society classification [10]. Given that many diseases could mimic AIP, multidisciplinary diagnosis, which requires a combination of clinical, radiological, and pathological findings, is needed. The case reported a patient with typical appearances of AIP. Apart from supplemental oxygen and mechanical ventilation, the patient received high-dose intravenous methylprednisolone for 5 d and normal dose of methylprednisolone for several days. However, the treatment could not contribute to the patient’s survival. The data from this case confirmed the occurrence of EMT in AIP and maybe beneficial for the treatment of this disease.

The process of lung injury with subsequent development of scar tissue in idiopathic pulmonary fibrosis has been likened to an abnormal wound healing model. A similar construct may be applied to AIP [11]. In theory, intervention before the deposition of mature collagen should allow the restoration of normal lung architecture. The marked expansion of myofibroblast numbers within the alveolar septa responds for the subsequent collagen production in the proliferative and fibrotic AIP [12]. Consistent with this conclusion, we also detected an amount of myofibroblast in the patient’s lung tissue. This myofibroblast may be related to the AIP process.

Previous observations revealed that injured epithelial cells could gradually lose their epithelial cell markers and polarity, thus expressing mesenchymal markers and acquiring single-cell motility [13], which was defined as EMT. During EMT, cytoskeletal reprogramming establishes the presence of α-SMA stress fibers in epithelial cells [14]. Through this transition, alveolar epithelial cells serve as an importance source of myofibroblasts during tissue injury response. Kalluri et al. [15] reported that under inflammatory stress, 30% of myofibroblasts can arise via EMT, whereas resident fibroblasts contribute only 23% in the kidney. Several articles reported that EMT may participate in various lung diseases, such as developmental disorders, fibrotic tissue remodeling, and lung cancer in humans [16-18]. Harada et al. [19] demonstrated the presence of EMT in patients with usual interstitial pneumonia pattern. However, Yamada et al. [20] obtained conflicting results and did not detect double-positive cells for E-cadherin, ICAM-1, LEA CD44v9, SP-A, α-SMA, or vimentin in lung tissues from patients with idiopathic pulmonary fibrosis and nonspecific interstitial pneumonia. Therefore, the evidence of myofibroblasts originating from epithelial cells through EMT in interstitial pneumonia remains controversial. Our ultrastructural data supported the existence of EMT in the lung tissue of patients with AIP. We also found that SPC and α-SMA, which are markers for alveolar epithelial cells and myofibroblasts, respectively [16], coexisted in the patient’s sections. The EMT program in epithelial cells is identified to be switched on by many transcription factors. For example, Snail, a major transcription factor governing EMT [21], could regulate the expression of genes related to epithelial and mesenchymal phenotype [22]. We found that the expression of Snail in the lung tissue of the patient was upregulated in the proliferative phase of AIP compared with the exudative phase of AIP.

In conclusion, the results of this study confirmed that alveolar epithelial cells underwent EMT, which maybe an important origin of myofibroblasts in the progression of AIP. Although pathological manifestation may vary from one case of AIP to another, our finding partly indicated the possibility and importance of EMT in AIP and provided a potential therapeutic method of preventing EMT in AIP.

### Consent

Written informed consent was obtained from the lung of the patient for publication of this Case Report and any accompanying images. A copy of the written consent is available for review by the Editor-in-Chief of this journal form.

## Abbreviations

AIP: Acute interstitial pneumonia; EMT: Epithelial-mesenchymal transition; HRCT: High-resolution computed tomography; DAD: Diffuse alveolar damage; SPC: Surfactant associated protein; α-SMA: α-Smooth muscle actin.

## Competing interests

The authors declare that they have no competing interests.

## Authors’ contributions

HL and JZ analyzed, interpreted the patient’s data and drafted the manuscript. XS performed the ultrastructural study and analysis. TW and ZL examined the patient and contributed to manuscript preparation. DH and XW contributed to discussions about the patient. CM performed immunohistochemical analysis. QZ and CL revised the pathology data and supervised the case report. All authors read and approved the final manuscript.

## Pre-publication history

The pre-publication history for this paper can be accessed here:

http://www.biomedcentral.com/1471-2466/14/67/prepub
